# Clinical utility in infants with suspected monogenic conditions through next‐generation sequencing

**DOI:** 10.1002/mgg3.684

**Published:** 2019-04-09

**Authors:** Sha Hong, Li Wang, Dongying Zhao, Yonghong Zhang, Yan Chen, Jintong Tan, Lili Liang, Tianwen Zhu

**Affiliations:** ^1^ Department of Neonatal Medicine Xin‐Hua Hospital Affiliated to Shanghai Jiao Tong University School of Medicine Shanghai China; ^2^ Department of Endocrinology and Genetic Metabolism Xin‐Hua Hospital Affiliated to Shanghai Jiao Tong University School of Medicine Shanghai China

**Keywords:** clinical utility, next‐generation sequencing, TRS, WES

## Abstract

**Background:**

Rare diseases are complex disorders with huge variability in clinical manifestations. Decreasing cost of next‐generation sequencing (NGS) tests in recent years made it affordable. We witnessed the diagnostic yield and clinical use of different NGS strategies on a myriad of monogenic disorders in a pediatric setting.

**Methods:**

Next‐generation sequencing tests are performed for 98 unrelated Chinese patients within their first year of life, who were admitted to Xin Hua Hospital, affiliated with Shanghai Jiao Tong University School of Medicine, during a 2‐year period.

**Results:**

Clinical indications for NGS tests included a range of medical concerns. The mean age was 4.4 ± 4.2 months of age for infants undergoing targeting specific (known) disease‐causing genes (TRS) analysis, and 4.4 ± 4.3 months of age for whole‐exome sequencing (WES) (*p* > 0.05). A molecular diagnosis is done in 72 infants (73.47%), which finds a relatively high yield with phenotypes of metabolism/homeostasis abnormality (HP: 0001939) (odds ratio, 1.83; 95% CI, 0.56–6.04; *p* = 0.32) and a significantly low yield with atypical symptoms (without a definite HPO term) (odds ratio, 0.08; 95% CI, 0.01–0.73; *p* = 0.03). TRS analysis provides molecular yields higher than WES (*p* = 0.01). Ninety‐eight different mutations are discovered in 72 patients. Twenty‐seven of them have not been reported previously. Nearly half (43.06%, 31/72) of the patients are found to carry 11 common disorders, mostly being inborn errors of metabolism (IEM) and neurogenetic disorders and all of them are observed through TRS analysis. Eight positive cases are identified through WES, and all of them are sporadic, of highly variable phenotypes and severity. There are 26 patients with negative findings in this study.

**Conclusion:**

This study provides evidence that NGS can yield high success rates in a tertiary pediatric setting, but suggests that the scope of known Mendelian conditions may be considerably broader than currently recognized.

## 1 INTRODUCTION

Rare disease is a health condition that affects a small number of people compared with other prevalent diseases in the general population (Baldovino, Moliner, Taruscio, Daina, & Roccatello, 2016). To date, between 5,000 and 8,000 distinct rare diseases have been documented with new ones reported regularly in the medical literature (Taruscio, Floridia, Salvatore, Groft, & Gahl, 2017). Although they are characterized by their rarity, the total number of patients affected is large [e.g., 25–50 millions in the United States (Fernandez‐Marmiesse, Gouveia, & Couce, 2018), 27–36 millions in the EU (Moliner & Waligora, 2017), and 16.8 millions in China (Yang, Su, Lee, & Bai, 2015)]. Rare diseases are typically severe, mostly genetic in origin, and the majority of cases are reported in patients with very early onset (Luzzatto et al., 2015). Therefore, efforts have been made continuously to identify the causative mutations for these infantile‐onset rare Mendelian diseases (Bacchelli & Williams, 2016), which is of great importance for patient management (Silibello et al., 2016) and family counseling (Babac, 2017).

Although traditional gene mapping approaches, such as Sanger sequencing (Botstein & Risch, 2003), linkage analysis (Teare & Santibanez Koref, 2014), and homozygosity mapping (Lander & Botstein, 1987) have led to great insights into Mendelian diseases over the past few decades; they are unable to detect all forms of variation in a single experiment. The rapid development of next generation sequencing (NGS) constituted a turning point for the advancement of our understanding of this type of diseases, which requires a broad search for causal variants across their genetically heterogeneous spectrum within a short time (Shen, Lee, Shen, & Lin, 2015), especially for life‐threatening or chronically debilitating cases. Today, different NGS techniques can be used for diagnostic purposes. Targeting specific (known) disease‐causing genes (TRS), which is applied to assist with molecular diagnosis of well‐defined disorders caused by a group of genes (Deleye, Gansemans, De Coninck, Van Nieuwerburgh, & Deforce, 2018) and sequencing the exons of every protein‐coding gene (whole‐exome sequencing: WES) for patients without an identified molecular cause are the two commonly used tools (Al‐Shamsi, Hertecant, Souid, & Al‐Jasmi, 2016).

In the present work, we study 98 patients with the clinical suspicion of a rare Mendelian disease with infantile onset. The patients were referred for NGS testing to establish a definitive genetic diagnosis. We demonstrate the clinical utility of NGS techniques in a pediatric setting by systematically describing our patient cohort.

## 2 MATERIALS AND METHODS

### 2.1 Editorial policies and ethical considerations

We have submitted our research proposal to the Ethics Committee of Xinhua Hospital affiliated to Shanghai Jiao Tong University School of Medicine. Our study protocol as well as the application form was fully reviewed and the organization has certified that this study would not incur any patient risk issues and is in accordance with the Declaration of Helsinki.

### 2.2 Clinical samples

Our study included 98 unrelated Chinese pediatric patients within the age range of 1 year or younger at the time of testing from Xin Hua Hospital affiliated with Shanghai Jiao Tong University School of Medicine between January 2016 and December 2017. They were referred by medical specialists for either WES or TRS, and have had the analysis and results disclosure completed. The patients in this cohort have diverse clinical features which are summarized in Tables 1, 2, 3. Informal written consent was obtained from the patients’ parents or legal guardians participating in the study prior to collecting 3 ml of the said patients’ peripheral blood.

**Table 1:** Variants of unknown significance, which were likely pathogenic

Gene^a^	Case ID∕gender	Age at testing (months)	Primary disease classification by HPO top‐level term	Variants	Segregation	SIFT_Predict§	PolyPhen_2_Predict§	Molecular Diagnosis (OMIM)
Autosomal dominant inheritance								
*SYNGAP1*	23∕Male	11	Abnormality of the nervous system	**c.2153T>G** (p.L718R)	Father:WT Mother:WT De novo	Damaging	Possibly damaging	Mental retardation (612621)
*NRAS*	97∕Male	11	Abnormality of the immune system	**c.35G>A** † (p.G12D)	Father: WT Mother:WT De novo	Damaging	Possibly damaging	Noonan syndrome6 (613224)
*KIF11*	144∕Female	16 days*	Abnormality of the eye	**c.77+1G>A**	Father: WT Mother:WT De novo	—	—	Microcephaly with or without chorioretinopathy, lymphedema, or mental retardation (152950)
*JAG1*	205∕Female	2	Abnormality of the digestive system	**c.2922dupT** (p.T975fs)	Father: WT Mother:WT De novo	—	—	Alagille syndrome 1 (118450)
*CLCN7*	389∕Female	11	Abnormality of the skeletal system	**c.C1700T** (p.T567T)	Father:WT Mother:WT De novo	Damaging	Probably damaging	Osteopetrosis, autosomal dominant 2 (166600)
*ABCC8*	503∕Female	1	Abnormality of the metabolism /homeostasis	**c.1671+2T>C**	Father: Het	—	—	Familial hyperinsulinemic hypoglycemia (256450)
Autosomal recessive inheritance								
*ARSB*	572∕Female	12	Abnormality of the skeletal system	**c.317G>C** (p.R106P) **c.264G>T** (p.Q88H)	Father: Het Mother:Het	Damaging Damaging	Possibly damaging Probably damaging	Mucopolysaccharidosis VI (253200)
*TGM1*	629∕Female	4 days*	Abnormality of the integument	**c.1130G>A** (p.C377Y) **c.871G>A** † (p.G291S) (PMID:19262603)	Father: Het Mother:Het	Damaging	Possibly damaging	Ichthyosis (242300)
*ATP6V0A4*	641∕Female	1.5	Abnormality of the genitourinary system	**c.639+1G>A**	Father: Het Mother:Het	‐	‐	Renal tubular acidosis, distal, autosomal recessive (602722)
*F13A1*	772∕Male	19 days*	Abnormality of the blood and blood‐forming tissues	**c.2015G>A** (p.G672E) **c.1352_1353del **(p.H451Rfs*29)	Unknown (Proband only)	Damaging	Probably damaging	Factor XIIIA deficiency (613225)
*ABCG8*	823∕Male	1	Abnormality of the digestive system	**c.786C>A** † (p.N262K) **c.1494_1495insGGGGATCTCG **(p.E500Dfs*105)	Father: Het Mother:Het	Damaging	Probably damaging	Sitosterolemia (210250)
*RYR1*	914∕Female	8	Abnormality of the musculature	**c.9161_9164delTCTC** (p.F3057Gfs*23) **c.14003C>G **(p.P4668R)	Father: Het Mother:Het	Damaging	Probably damaging	Minicore myopathy with external ophthalmoplegia (255320)
*TPP2*	991∕Male	12	Abnormality of the immune system	**c.229A>C** † (p.N77H) **c.1361A>G** † (p.N454S)	Father: Het Mother:Het	Damaging Damaging	Possibly damaging Possibly damaging	Tripeptidyl‐peptidase II deficiency (190470)
X‐linked inheritance								
*IL2RG*	1036∕Male	12	Abnormality of the immune system	**c.943_962del** (p.K315Afs*6)	Inherited hemi	‐	‐	X‐linked severe combined immunodeficiency (300400)
*NDP*	1193∕Male	12	Abnormality of the eye	**c.320_353del** (p.R107fs)	Inherited hemi	‐	‐	Norrie disease (310600)

Note

Abbreviations: HPO, human phenotype ontology; OMIM, Phenotype Mendelian Inheritance in Man.

Variants in bold were unreported previously.

If SIFTori is smaller than 0.05 (rank score >0.395) the corresponding nsSNV is predicted as “Damaging”; otherwise it is predicted as “Tolerated”. Multiple predictions separated by “;”

Polyphen2 prediction based on HumDiv, “D” (“probably damaging”, HDIV score in [0.957, 1] or rank score in [0.52844, 0.89865]), “P” (“possibly damaging,” HDIV score in [0.453, 0.956] or rank score in [0.34282, 0.52689]), and “B” (“benign”, HDIV score in [0, 0.452] or rank score in [0.02634, 0.34268]).

a *SYNGAP1*: NM_006772.2; *NRAS*: NM_002524.5; *KIF11*: NM_004523.4; *JAG1*: NM_000214.3; *CLCN7*: NM_001287.6; *ABCC8*: NM_000352.4; *ARSB*: NM_000046.5; *TGM1*: NM_000359.3; *ATP6V0A4*: NM_020632.3; *F13A1*: NM_000129.3; *ABCG8*: NM_022437.3; *RYR1*: NM_000540.2; *TPP2*: NM_003291.4; *IL2RG*: NM_000206.2; *NDP*: NM_000266.4.

* Less than 1 month

† Mutations that have been reported their pathogenicity previously and referred by their PMID number by a review of the literature and variant databases such as ClinVar and ExAC Browser (Beta).

§ SIFT and PolyPhen‐2 are two pathogenicity predictions used to evaluate putative pathogenicity of novel nonsynonymous coding variants (unreported previously).

^‐^ All variants, including rearrangements, stop codon‐introducing (nonsense), insertion/deletion (indel), and splice site ones were regarded as null alleles, abolishing production of the corresponding protein from the affected allele.

**Table 2:** Summary of patients with variants belonging to category I

Gene^a^	Case ID∕gender	Age at testing (months)	Primary disease classification by HPO top‐level term	Variants (PMID)‡	Segregation	Molecular diagnosis (OMIM)
Autosomal dominant inheritance						
*COL1A1*	54∕Female	8	Abnormality of the skeletal system	c.432dupC (p.G145fs) (22753364)	Father: Het	Osteogenesis imperfecta, type I (166200)
*COL1A2*	59∕Female	10	Abnormality of the skeletal system	c.3305G>A (p.G1102D) (17078022)	Father: WT Mother: Unknown	Osteogenesis imperfecta, type II/Osteogenesis imperfecta, type IV (166200)
COL2A1	121∕Male	1	Abnormality of prenatal development or birth	c.2582G>T (p.G861V) (23653587)	Father: WT; Mother: WT De novo	SMED Strudwick type (184250)
*KCNJ11*	186∕Female	1	Abnormality of metabolism/homeostasis	c.602G>A (p.R201H) (15115830)	Father: WT; Mother: WT De novo	Diabetes mellitus, transient neonatal (610582)
*NF1*	247∕Female	6	Abnormality of the nervous system	**DelE28−29** (p.D1237Vfs*8) (26189818)	Unknown (Proband only)	Neurofibromatosis, type 1 (162200)
	293∕Female	8	Abnormality of the nervous system	Multiple exons del (p.?) (26740943)	Unknown (Proband only)	
	332∕Female	1	Abnormality of the nervous system	c.647T>C (p.L216P) (10712197)	Father: WT; Mother: WT De novo	
	361∕Female	12	Abnormality of the nervous system	c.6841C>T (p.Q2281X) (8837715)	Father: WT; Mother: WT De novo	
*RB1*	390∕Male	3	Abnormality of the eye	Ex 1–21 del (p.?) (23301675)	Unknown (Proband only)	Retinoblastoma (180200)
	433∕Male	12	Abnormality of the eye	**c.2285_2286delAG** (p.R763Tfs*31)^‐^	Father: WT; Mother: WT De novo	
*CHD7*	447∕Female	20 days*	Abnormality of prenatal development or birth	c.6217C>T (p.Q2073X) (29304373)	Father: WT; Mother: WT De novo	CHARGE syndrome (214800)
*ABCC8*	459∕Female	4	Abnormality of metabolism/homeostasis	**c.3752G**>**C** (p.R1251P)§,^‐^ (Damaging &Probably damaging)	Father: Het	Hyperinsulinemic hypoglycemia, familial, 1 (256450)
*ANK1*	519∕Female	5	Abnormality of the blood and blood‐forming tissues	**c.2101G**>**T **(p.G701X)^‐^	Mother: Het	Spherocytosis, type 1 (182900)
*JAG1*	526∕Male	2	Abnormality of the digestive system	**c.2347delA** (p.T783Pfs*37)^‐^	Father: WT; Mother: WT De novo	Alagille syndrome 1 (118450)
*ATP2A2*	531∕Male	8	Abnormality of the integument	**c.2832_2833del** (p.L945Dfs*36)^‐^	Unknown (Proband only)	Acrokeratosis verruciformis (101900)
Autosomal recessive inheritance						
*CPS1*	593∕Male	3 days*	Abnormality of metabolism/homeostasis	c.446T>C (p.L155S) (21120950) c.2023del T (p.C1132Afs*3) (21120950)	Father: Het Mother: Het	Carbamoylphosphate synthetase I deficiency (237300)
*GJB2*	625∕Male	4	Abnormality of the ear	c.299_300del (p.H100Rfs*14) (10633133) c.235delC (P.L79Cfs*3) (10501520)	Unknown (Proband only)	Deafness, autosomal recessive 1A (220290)
	658∕Female	2	Abnormality of the ear	c.235delC (p.L79fs) (22952768)	Father: Het Mother: Het	Deafness, autosomal recessive 1A (220290)
*GSS*	684∕Male	6 days*	Abnormality of metabolism/homeostasis	c.738dupG (p.S247Vfs*59) (15717202) Ex 3 dup (p.?) (15717202)	Father: Het Mother: Het	Glutathione synthetase deficiency (266130)
*ITGB2*	695∕Male	1	Abnormality of the immune system	*c*.*817G*>*A*（p.G273R) (9884339)	Paternal UPD	Leukocyte adhesion deficiency (116920)
*MMACHC*	711∕Male	2	Abnormality of metabolism/homeostasis	c.217C>T（p.R73X）(16311595) c.609G>A（p.W203X）(16311595)	Father: Het Mother: Het	Methylmalonic aciduria and homocystinuria, cblC type (277400)
	734∕Male	5	Abnormality of metabolism/homeostasis	c.394C>T（p.R132X）(16311595) c.457C>T（p.R153X）(16311595)	Father: Het Mother: Het	
*MUT*	739∕Male	2	Abnormality of metabolism/homeostasis	c.729_730insTT (p.D244Lfs*39) (16281286) c.398_399del (p.G133Vfs*6) (23430940)	Father: Het Mother: Het	Methylmalonic aciduria, mut(0) type (251000)
	780∕Male	1	Abnormality of metabolism/homeostasis	c.1880A>G (p.H627R) (10923046) c.2179C>T ( p.R727X) (16281286)	Father: Het Mother: Het	
	822∕Female	2	Abnormality of metabolism/homeostasis	*c*.*755dupA* (p.H252Qfs*6) (23430940)	Father: Het Mother: Het	
	879∕Female	16 days*	Abnormality of metabolism/homeostasis	c.1106G>A (p.R369H) (9285782) c.349G>T (p.E117X) (7951229)	Father: Het Mother: Het	
*PAH*	883∕Male	23 days*	Abnormality of metabolism/homeostasis	c.770G>T (p.G257V) (11360625) c.977G>A (p.W326X) (1301187)	Father: Het Mother: Het	Phenylketonuria (261600)
	939∕Male	1	Abnormality of metabolism/homeostasis	c.442−1G>A (p.IVS4−1G>A) (1998345) c.116_118delTCT (p.39_40del) (8406445)	Father: Het Mother: Het	
*PCSK1*	947∕Male	27 days*	*N*	*c*.*1777G*>*A* (p.G593R) (9207799)	Father: Het Mother: Het	Obesity with impaired prohormone processing (600955)
*RAG1*	966∕Male	9	Abnormality of the immune system	*c*.*2923C*>*T* (p.R975W) (18463379)	Father: Het Mother: Het	Severe combined immunodeficiency, B cell‐negative (601457)
*SLC26A4*	985∕Female	8	Abnormality of the ear	c.754T>C (p.S252P) (12676893) c.1229C>T (p.T410M) (9618167)	Unknown (Proband only)	Deafness, autosomal recessive 4, with enlarged vestibular aqueduct (600791)
*SPINK5*	1006∕Male	6	Abnormality of the integument	c.2459dupA (p.K824Efs*4) (10835624) c.2459delA (p.K823Rfs*119) (11841556)	Father: Het Mother: Het	Netherton syndrome (256500)
*TYR*	1014∕Female	8	Abnormality of the integument	c.896G>A (p.R299H) (1642278) c.926dupC (p.T309fs) (2517365)	Father: Het Mother: Het	Albinism, oculocutaneous, type IA (203100)
*GTPBP3*	1033∕Female	1.4	Abnormality of the metabolism/homeostasis	**c.253C**>**T** (p.R85X)^‐^ **c.479C**>**T** (p.A160V)§ (Damaging &Probably damaging)	Father: Het Mother: Het	Combined oxidative phosphorylation deficiency 23 (616198)
*ABCC8*	1057∕Male	1	Abnormality of metabolism/homeostasis	c.2351T>G (p.V784G) (17668386) c.3124_3126delACCinsCAGCCAGGAACTG (p.T1043Qfs) (17668386)	Father: Het Mother: Het	Hyperinsulinemic hypoglycemia,familial, 1 (256450)
*MAT1A*	1064∕Male	1	Abnormality of the metabolism/homeostasis	c.181A>C (p.K61Q) (15569761) c.1067G>C (p.R356P) (15569761)	Father: Het Mother: Het	Methionine adenosyltransferase deficiency (250850)
*NPC1*	1069∕Male	6	Abnormality of metabolism/homeostasis	c.1484T>C (p.L495P) (15774455) c.3634G>T (p.V1212L) (15774455)	Father: Het Mother: Het	Niemann‐Pick disease type C1 (257220)
*IL10RA*	1082∕Female	12	Abnormality of the digestive system	c.299T>G (p.V100G) (22476154) c.301C>T (p.R101W) (22476154)	Father: Het Mother: Het	Inflammatory bowel disease 28 (613148)
*SMN1*	1103∕Male	7	Abnormality of the nervous system	**DelE8−13** (p.?)^‐^	Unknown (Proband only)	Spinal muscular atrophy‐1 (253300)
*C7*	1107∕Male	4	Abnormality of the immune system	**c.830G**>**C** (p.W277S)§ (Damaging &Possibly damaging) **c.1258A**>**C** (p.K420Q)	Father: Het Mother: Het	C7 deficiency (610102)
*PEX26*	1124∕Male	2	Abnormality of the digestive system	**c.29delC** (p.L12Sfs*70)^‐^ **c.359T**>**G** (p.V120G)§ (Damaging &Probably damaging)	Father: Het Mother: Het	Peroxisome biogenesis disorder 7A (Zellweger) (614872)
*SRD5A2*	1131∕Male	2	Abnormality of the genitourinary system	c.623C>T (p.T208I) (8784107) c.680G>A (p.Arg227Gln) (8784107)	Father: Het Mother: Het	Pseudovaginal perineoscrotal hypospadias (264600)
X‐linked inheritance						
*ABCD1*	1139∕Male	11	Abnormality of the nervous system	**c.1553G**>**A** (p.R518Q)	Inherited hemi	Adrenoleukodystrophy (300100)
*CYBB*	1158∕Male	2	Abnormality of the immune system	**DelE1−13** (p.?)	de novo hemi	Chronic granulomatous disease, X‐linked (306400)
*DMD*	1172∕Male	1	Abnormality of the nervous system	**DupE28−43 **(p.?) (25482253)	de novo hemi	Duchenne/Becker muscular dystrophy (310200)
	1198∕Male	1.5	Abnormality of the nervous system	**DelE8−9 (c.650‐?_960 + ?del)** (22894145)	Inherited hemi	
	1201∕Male	1.5	Abnormality of the nervous system	**DelE8−9 (c.650‐?_960 + ?del)** (22894145)	Inherited hemi	
	1206∕Male	1	Abnormality of the nervous system	c.8713C>T (p.R2905X) (7611292)	Inherited hemi	
	1221∕Male	1	Abnormality of the nervous system	**DelE45−52(c.6439‐?_7660+?del)** (26911353)	Inherited hemi	
	1223∕Male	9	Abnormality of the nervous system	**DelE8−13** (p.?)^‐^	Inherited hemi	
*IKBKG*	1239∕Female	10	Abnormality of the eye	c.184C>T (p.R62X)	Inherited hemi	Incontinentia pigmenti (308300)
*IL2RG*	1244∕Male	4	Abnormality of the immune system	c.854+2T>C (p.?) (10794430)	Inherited hemi	X‐linked severe combined immunodeficiency (300400)
*NDP*	1257∕Male	19 days*	Abnormality of the eye	Nullizygous del whole gene (p.NDPdel) (22382802)	Inherited hemi	Norrie disease (ND) (310600)
*OTC*	1273∕Male	2	Abnormality of metabolism/homeostasis	c.540G>C (p.Q180H) (9452024)	Inherited hemi	Ornithine transcarbamylase deficiency (311250)
*RS1*	1298∕Male	60	Abnormality of the eye	c.522+1G>A (p.?) (12920343)	Inherited hemi	Retinoschisis (312700)
Isolated cases						
*SNRPN*	1311∕Female	12	*N*	15q Mat del (P.?) (10802660)	Inherited hemi	PraderWilli syndrome (176270)
Mitochondrial inheritance						
*MTND5*	1326∕Female	8	Abnormality of the nervous system	c.G13513A (p.D393N) (9299505)	Mother: WT De novo	MELAS (Mitochondrial Encephalomyopathy, Lactic Acidosis, And Stroke‐Like Episodes）(540000)

Note

Abbreviations: PMID, PubMed Identifier; OMIM, Phenotype Mendelian Inheritance in Man; UPD, uniparental disomy; HPO, Human Phenotype Ontology; *N*, Patients had clinical features of more than two of the broad aforementioned HPO term or atypical symptoms so that they were not given the exact HPO terms for their primary phenotypes.

Variants in bold were unreported previously.

For variants with autosomal recessive inheritance, homozygous variants are in Italics.

If SIFTori is smaller than 0.05 (rank score >0.395) the corresponding nsSNV is predicted as “Damaging”; otherwise it is predicted as “Tolerated.” Multiple predictions separated by “;”

Polyphen2 prediction based on HumDiv, “D” (“probably damaging,” HDIV score in [0.957, 1] or rank score in [0.52844, 0.89865]), “P” (“possibly damaging,” HDIV score in [0.453, 0.956] or rank score in [0.34282, 0.52689]), and “B” (“benign,” HDIV score in [0, 0.452] or rank score in [0.02634, 0.34268]).

a *COL1A1*: NM_000088.3; *COL1A2*: NM_000089.3; *COL2A1*: NM_001844.5; *KCNJ11*: NM_000525.3; *NF1*: (NM_000267.3); RB1: NM_000321.2; *CHD7*: NM_017780.4; *ABCC8*: NM_000352.4; *ANK1*: NM_000037.3; *JAG1*: NM_000214.3; *ATP2A2*: NM_001681.3; *CPS1*: NM_001122633.2; *GJB2*: NM_004004.6; *GSS*: NM_000178.4; *ITGB2*: NM_000211.5; *MMACHC*: NM_015506.3; *MUT*: NM_000255.4; *PAH*: NM_000277.3; *PCSK1*: NM_000439.5; *RAG1*: NM_000448.2; *SLC26A4*: NM_000441.1; *SPINK5*: NM_006846.3; *TYR*: NM_000372.5; *GTPBP3*: NM_032620.4; *MAT1A*: NM_000429.3; *NPC1*: NM_000271.5; *IL10RA*: NM_001558.3; *SMN1*: NM_000344.3; *C7*: NM_000587.3; *PEX26*: NM_017929.5; *SRD5A2*: NM_000348.4; *ABCD1*: NM_000033.4; *CYBB*: NM_000397.3; *DMD*: NM_004006.2; *IKBKG*: NM_001099856.4; *IL2RG*: NM_000206.2; *NDP*: NM_000266.4. *OTC*: NM_000531.6; *RS1*: NM_000330.3; *SNRPN*: NM_022806.4; *MTND5*: YP_003024036.1.

* less than 1 month

‡ Variants that have been reported their pathogenicity previously and/or referred by their PMID number by a review of the peer‐reviewed literature and variant databases such as ClinVar and ExAC Browser (Beta).

^‐^ All variants, including rearrangements, stop codon‐introducing (nonsense), insertion/deletion (indel), and splice site ones were regarded as null alleles, abolishing production of the corresponding protein from the affected allele.

§ SIFT and PolyPhen‐2 are two pathogenicity predictions used to evaluate putative pathogenicity of novel nonsynonymous coding variants (unreported previously).

^†^ The molecular diagnoses were obtained by Multiplex ligation‐dependent probe amplification (MLPA) analysis.

**Table 3:** Negative diagnosis by NGS tests in 26 individuals

Case ID	Primary disease classification by HPO top‐level term	Gender	Age at testing (months)	Comments
37	Abnormality of the metabolism/homeostasis	Male	22 days*	The biochemical findings and phenotypes were consistent with HMG‐CoA lyase deficiency [OMIM: 246450] with a recessive inheritance pattern, but only one variant (c.122G>A (p.R41Q)) which was reported previously to be associated with the disorder was found in *HMGCL* gene
71	*N*	Male	1.5	No pathogenic variants related to patient phenotypes were identified
129	Abnormality of the integument	Female	9	No pathogenic variants related to patient phenotypes were identified
173	Abnormality of the cardiovascular system	Female	8 days*	No pathogenic variants related to patient phenotypes were identified
212	Abnormality of the nervous system	Male	12	No pathogenic variants related to patient phenotypes were identified
299	*N*	Female	15 days*	No pathogenic variants related to patient phenotypes were identified
374	Abnormality of the metabolism/homeostasis	Female	16 days*	The biochemical findings and phenotypes were consistent with Coenzyme Q10 deficiency [OMIM: 607426] with a recessive inheritance pattern, but only one variant (c.170_171insTGGGCTCGCGAGCCGC (p.F59Lfs*39)) which was predicted as a null allele was found in *COQ2* gene
412	*N*	Female	5 days*	No pathogenic variants related to patient phenotypes were identified
471	Abnormality of the blood and blood‐forming tissues	Male	18 days*	No pathogenic variants related to patient phenotypes were identified
524	Abnormality of the endocrine system	Male	23 days*	The biochemical findings and phenotypes were consistent with thyroid dyshormonogenesis [OMIM: 274500] with a recessive inheritance pattern, but only one variant (c.2654G>T (p.R885L)) which was previously reported to be associated with the disorder was found in *DUOX2* gene
550	Abnormality of the integument	Female	3	No pathogenic variants related to patient phenotypes were identified
575	Abnormality of the nervous system	Male	1	No pathogenic variants related to patient phenotypes were identified
621	Abnormality of the nervous system	Male	2.5	No pathogenic variants related to patient phenotypes were identified
662	Abnormality of prenatal development or birth	Female	3	No pathogenic variants related to patient phenotypes were identified
707	Abnormality of the nervous system	Female	11	No pathogenic variants related to patient phenotypes were identified
756	Abnormality of the nervous system	Male	10	No pathogenic variants related to patient phenotypes were identified
797	Abnormality of the genitourinary system	Male	8	No pathogenic variants related to patient phenotypes were identified
854	Abnormality of the metabolism/homeostasis	Male	24 days*	The biochemical findings and phenotypes were consistent with Carnitine deficiency [OMIM: 212140] with a recessive inheritance pattern, but only one variant (c.51C>G (p.F17L)) which was previously reported to be associated with the disorder was found in *SLC22A5* gene
902	Abnormality of the nervous system	Male	4	The VUS (c.817C>T (p.Q273X) in *ATP13A4* gene) that is predicted as a null allele explains several of the clinical features (seizures and epilepsy) of the patient
941	Abnormality of the nervous system	Female	10	The phenotypes and familial (segregation) results were consistent with mental retardation, autosomal recessive, 37 [OMIM 615493], but one VUS (c.8988G>C (p.Q2996H) in *ANK3* gene) is predicted consistently as un‐damaging (Tolerated for SIFT§ and Benign for PolyPhen_2§)
978	Abnormality of the eye	Female	11	The phenotypes and familial (segregation) results were partly consistent with Cohen syndrome [OMIM: 216550] with a recessive inheritance pattern, but two VUS (c.10333G>A (p.V3445M) and c.10718C>T (p.T3573I) in *VPS13B* gene) are both predicted consistently as un‐damaging (Tolerated for SIFT§ and Benign for PolyPhen_2§)
1003	Abnormality of the cardiovascular system	Male	1.3	This patient received triple molecular diagnoses. The VUS (c.89delA (p.D30fs) in *ACTN2* gene) that is predicted as a null allele explains most of the clinical features of the patient to be diagnosed with Cardiomyopathy, hypertrophic, 23, with or without LVNC [OMIM: 612158] with a dominant inheritance pattern; the VUS (c.439C>T (p.L147F) in *JUP* gene that is predicted consistently as damaging (Damaging for SIFT§ and Probably damaging for PolyPhen_2§) explains most of the clinical features of the patient to be diagnosed with Arrhythmogenic right ventricular dysplasia 12 [OMIM: 611528] with a dominant inheritance pattern; the VUS (c.103G>C (p.G35R) in *LMNA* gene that is predicted consistently as damaging (Damaging for SIFT§ and Probably damaging for PolyPhen_2§) explains most of the clinical features of the patient to be diagnosed with Cardiomyopathy, dilated, 1A [OMIM: 115200] with a dominant inheritance pattern
1041	Abnormality of the nervous system	Female	1	The phenotypes were consistent with Mental retardation, autosomal recessive 38 [OMIM: 615516], but one VUS (c.8329A>G (p,M2777V) in *HERC2* gene) is predicted consistently as un‐damaging (Tolerated for SIFT and Benign for PolyPhen_2); another VUS (c.5213G>C (p.W1738S) in *HERC2* gene) is predicted inconsistently (Damaging for SIFT§and Benign for PolyPhen_2§)
1073	Abnormality of the metabolism/homeostasis	Male	4 days*	The phenotypes and familial (segregation) results were consistent with Ornithine transcarbamylase deficiency [OMIM: 311250] with a X‐linked inheritance pattern, but the VUS (c.176T>C (p.L59P) in *OTC* gene) is predicted inconsistently (Tolerated for SIFT§ and Probably damaging for PolyPhen_2§)
1109	Abnormality of the nervous system	Female	12	The phenotypes and familial (segregation) results were consistent with Spastic paraplegia 39, autosomal recessive [OMIM: 612020], but one VUS (c.2096G>A (p.S699N) in *PNPLA6* gene) is predicted inconsistently (Damaging for SIFT§ and Benign for PolyPhen_2§)
1329	Abnormality of the integument	Female	2	This patient received dual molecular diagnoses. The VUS (c.5124+1G>T in *COL7A1* gene) that is predicted as a null allele explains most of the clinical features of the patient to be diagnosed with Epidermolysis bullosa dystrophica [OMIM: 131750] with a dominant inheritance pattern; the VUS (c.2975G>C (p.C992S) in *RTEL1* gene that is predicted consistently as damaging (Damaging for SIFT§ and Probably damaging for PolyPhen_2§) explains most of the clinical features of the patient to be diagnosed with Dyskeratosis congenita, autosomal recessive 5 [OMIM: 615190] with a dominant inheritance pattern

Note

Abbreviations: HPO, human phenotype ontology; HP, human phenotype;VUS: variants of uncertain significance; OMIM, Phenotype Mendelian Inheritance in Man.

If SIFTori is smaller than 0.05 (rank score >0.395) the corresponding nsSNV is predicted as “Damaging”; otherwise it is predicted as “Tolerated”. Multiple predictions separated by “;”

Polyphen2 prediction based on HumDiv, “D” (“probably damaging,” HDIV score in [0.957, 1] or rank score in [0.52844, 0.89865]), “P” (“possibly damaging,” HDIV score in [0.453, 0.956] or rank score in [0.34282, 0.52689]), and “B” (“benign”, HDIV score in [0, 0.452] or rank score in [0.02634, 0.34268]).

* Less than one month

§ SIFT and PolyPhen‐2 are two pathogenicity predictions used to evaluate putative pathogenicity of novel nonsynonymous coding variants (unreported previously).

### 2.3 The targeting specific disease‐causing genes (TRS) analysis and Sanger confirmation

A total of 12 different specific disease panels based on Targeted Exome Sequencing (TES) (designed by MyGenostics, Beijing, China) were implemented on our cohort according to their clinical features to collect the protein‐coding regions of the targeted genes. A gene capture strategy with GenCap custom exome enrichment kits (MyGenostics, Beijing, China) was used in our study. The extracted DNA samples were quantified by Nanodrop 2000 (Thermo Fisher Scientific, Wilmington, DE). A minimum of 3 mg of DNA from the patient was used to generate index libraries (average size of 350–450 bp, including adapter sequences) for Solexa HiSeq2000 sequencing (Illumina, San Diego, CA). Sequencing was carried out using 90 cycles per read. The obtained mean exome coverage was more than 98%, with variants accuracy at more than 99%. Clinically relevant variants, from proband and parental samples (whenever available), were confirmed by Sanger sequencing.

For those patients with clinical suspicions of Duchenne/Becker muscular dystrophies (OMIM 310200), Neurofibromatosis, type 1 (OMIM 162200), Spinal muscular atrophy‐1 (OMIM 253300), and Prader‐Willi syndrome (OMIM 176270), we performed multiplex ligation‐dependent probe amplification (MLPA) analysis (Stuppia, Antonucci, Palka, & Gatta, 2012) to detect the deletion or duplication of *DMD* (MIM 300377), *NF1* (MIM 613113), *SMN1* (MIM 600354), and *SNRPN* (MIM 182279) genes in exons using the SALSA MS‐MLPA P034‐B2/P035‐B1 *DMD* (NM_004006.2), P081‐C1/P082‐C1 *NF1* (NM_000267.3), P060‐B2 *SMN1* (NM_000344.3), and ME028‐B2 Prader‐Willi/Angelman kits (MRC‐Holland, Amsterdam, The Netherlands) according to the manufacturer's instructions (MyGenostics, Beijing, China). The sample with a single exon deletion was further verified by PCR and direct sequencing. Patients with negative MLPA were further tested for small mutations.

### 2.4 Whole‐exome sequencing and Sanger confirmation

Whole‐exome sequencing and its analysis protocols were developed and validated by MyGenostics, Beijing, China. Genomic DNA from patients was fragmented by sonication. The fragments were ligated to illumina multiplexing paired‐end adapters, amplified by polymerase chain‐reaction assay, and hybridized to biotin‐labeled P039‐Exome (at 65°C for 16 hr). Paired‐end sequencing was performed on Illumina NextSeq 500 platform, with an average sequencing depth of more than 100. Meanwhile, coverage of the targeted base for the N20 read was 95%. Following sequencing, raw image files were processed using Bcl2Fastq software（Bcl2Fastq 2.18.0.12, Illumina, Inc.) for base calling and raw data generation. Low‐quality variations were filtered out using a quality score ≥20. Short Oligonucleotide Analysis Package (SOAP) aligner software (SOAP2.21; soap.genomics.org.cn/soapsnp.html) was then used to align and refresh reads to the reference human genome (hg19). Variants were prioritized on the basis of the phenotype‐driven gene lists for each participant and predicted effect. Clinically relevant variants, from proband and parental samples (whenever available), were confirmed by Sanger sequencing.

### 2.5 Molecular diagnoses

In this study, sequence changes including rearrangements, stop codon‐introducing (nonsense), insertion/deletion (indel) variants, and splice site variants were regarded as null alleles (Lander & Botstein, 1987), abolishing production of the corresponding protein from the affected allele. Pathogenicity prediction (Nakken, Alseth, & Rognes, 2007) (SIFT [sift.bii.a-star.edu.sg] and PolyPhen‐2 [genetics.bwh.harvard.edu]) were used to evaluate putative pathogenicity of novel nonsynonymous coding variants (unreported previously). All our findings are classified under three categories. We describe causative mutations in the context of their consistent correspondence to the patients’ phenotypes, biochemical findings, familial (segregation) studies, or previously reported pathogenicity, and group these patients accordingly into category I by following the American College of Medical Genetics and Genomics (ACMG) variant classification guidelines (Lander & Botstein, 1987). We indicate those variants which were consistent with patients’ phenotypes and had been predicted to be deleterious though unreported previously, patients with such features were grouped under category II. Patients with variants belonging to category I and II were identified as either positive or confirmed cases. Category III include the patients with variants which were inconsistent with patients’ phenotypes or biochemical/ familial (segregation) study results, as well as those with no identified pathogenic variants and those with previously unreported variants that were predicted as either consistently nondamaging or inconsistent between two prediction tools.

We used a human phenotype ontology (HPO) term (Shen et al., 2015) to classify the primary disease of the patient that can be annotated by his clinical notes, which is essential for variant interpretation in our cohort characteristic of clinically and genetically heterogeneous disorders.

### 2.6 Statistical analysis

A chi‐squared test was applied to compare the different diagnostic yields in the two groups of patients. The statistical calculations were performed using SPSS 22.0 version.

## 3 RESULTS

This work is a retrospective evaluation of an advanced clinical diagnostic tool utility in a tertiary pediatric center. In this work, we investigated the diagnostic yield of NGS in a cohort of 98 Chinese patients with suspected rare Mendelian disease of infantile onset. Their clinical and biochemical profiles were undertaken prior to the referral for NGS analysis.

The NGS method consisted of TRS analysis (*n* = 81/98, 82.65%) and WES (*n* = 17/98, 17.35%) depending on a range of clinical concerns. There was no significant difference in the age of the patients at the time of testing between the two categories (*p* = 0.9678). The median turnaround time of TRS analysis was 30.0 days and that of WES was 50.0 days. Consequently, the median (*SEM*) age of diagnosis in infants who were undergoing TRS analysis (mean ± *SD*: 4.4 ± 4.2 months of age) was not significantly younger or older than those who were undergoing WES (mean ± *SD*: 4.4 ± 4.3 months of age).

The NGS results of 98 patients were divided into the following groups depending on our method criteria. Group A included 15 patients in line with Category II, shown in Table 1. Group B included 57 patients in line with Category I, shown in Table 2, while Group C included 26 patients in line with Category III, shown in Table 3. Therefore, a definitive genetic diagnosis was achieved for 72 patients (73.47%, 72/98) in the study. The TRS analysis provided higher molecular yields for 64 of 81 pediatric patients (79.01%) than WES for 8 of 17 ones (47.06%) (OR: 0.24; 95% CI (0.08–0.70); *p*: 0.01, Fisher’s exact test). All reported pathogenic and deleterious point mutations in Tables 1 and 2, confirmed by Sanger sequencing.

### 3.1 Cohort description

All patients were under 1 year of age at the time of NGS analysis (average age was 4.38 months), with 41 females (41.84%, 41/98) and 57 males (58.16%, 57/98). Eighteen of them were <1 month of age (18.37%, 18/98), while 38 were between 1‐ and 3‐month‐old infants (38.78%, 38/98). It was shown that more than half of our patients developed various symptoms within 3 months of age.

**Figure 1:**
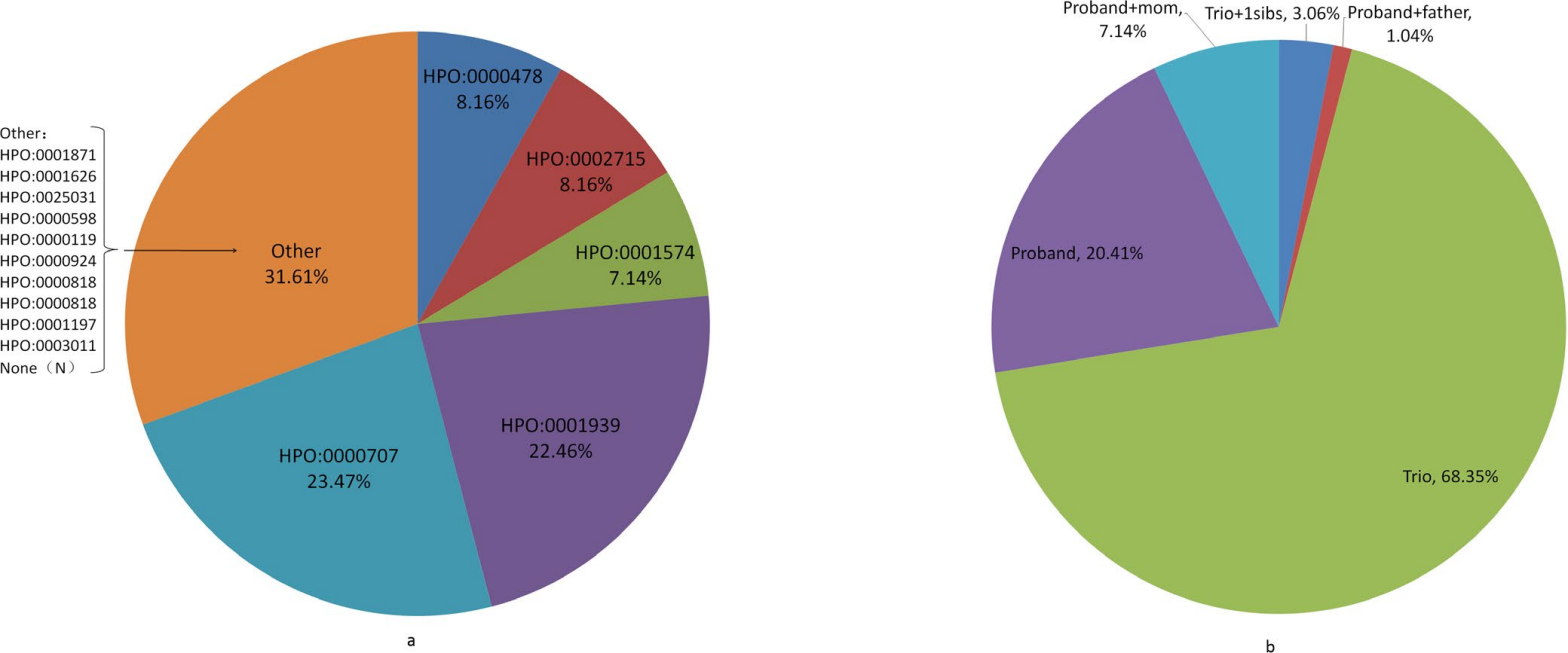
Descriptive statistics of the patient cohort. (a) Primary indication; (b) Family members tested. Abbreviations: HPO, human phenotype ontology; HP, human phenotype. HPO(0000119): Abnormality of the genitourinary system; HPO(0000478): Abnormality of the eye; HPO(0000598): Abnormality of the ear; HPO(0000707): Abnormality of the nervous system; HPO(0000818): Abnormality of the endocrine system; HPO(0000924): Abnormality of the skeletal system; HPO(0001197): Abnormality of prenatal development or birth; HPO(0001574): Abnormality of the integument; HPO(0001626): Abnormality of the cardiovascular system; HPO(0001871): Abnormality of the blood and blood‐forming tissues; HPO(0001939): Abnormality of the metabolism/homeostasis; HPO(0002715): Abnormality of the immune system; HPO(0003011): Abnormality of the musculature; HPO(0025031): Abnormality of the digestive system; None (N): Patients had clinical features of more than two of the broad aforementioned HPO term or atypical symptoms so that they were not given the exact HPO terms for their primary phenotypes

Of this cohort, 23.47%, 22.45%, 8.16%, 8.16%, and 7.14% were patients with primary phenotypes defined by HPO term related to abnormality of the nervous system (HP:0000707), abnormality of the metabolism/homeostasis (HP:0001939), abnormality of the immune system (HP:0002715), abnormality of the eye (HP:0000478), and abnormality of the integument (HP:0001574), respectively (Figure 1a, primary indication). 5.10% (5/98) had clinical features of more than two of the broad aforementioned HPO term or atypical symptoms so that they were not given the exact HPO terms for their primary phenotypes. For most patients, both parents’ DNA was tested (Figure 1b, family members tested).

### 3.2 Molecular diagnosis

Of the 98 probands, 72 carried 125 mutant alleles at 53 different chromosomal loci that satisfied the criteria for a confirmed molecular diagnosis (Tables 1 and 2). A diverse group of disorders was represented by patients who tested positive. Three diseases, namely Neurofibromatosis type 1 (OMIM 162200), Duchenne/Becker muscular dystrophies (OMIM 310200), and Methylmalonic aciduria mut (0) type (OMIM 251000), which were caused by variants in the *NF1*, *DMD,* and *MUT* (MIM 609058) genes, were observed in 14 diagnosed infants (19.44%, 14/72). They comprised the most frequent infantile onset single‐gene disorders in our cohort. Other disorders found in at least two infants included Alagille syndrome 1 (OMIM 118450), persistent hyperinsulinemic hypoglycemia of infancy (OMIM 256450), retinoblastoma (OMIM 180200), deafness autosomal recessive 1A (OMIM 220290), methylmalonic aciduria and homocystinuria cblC type (OMIM 277400), phenylketonuria (OMIM 261600), Norrie disease (OMIM 310600), severe combined immunodeficiency, and X‐linked (OMIM 300400), which collectively comprised 17 of 72 diagnoses (23.61%). Nearly half (43.06%, 31/72) of the diagnosed patients were identified to have the above 11 different disorders.

Ninety‐eight different mutations were discovered in 72 diagnosed patients and a full range of mutation types was observed, including 44 missense, 17 frame‐shift, 13 nonsense, 12 CNV (copy number variation), 6 in‐frame, and 6 spicing (Tables 1 and 2). Missense (44.90%, 44/98) and frame‐shift (17.35%, 17/98) mutations made up the highest percentages of changes. Moreover, 27 of the 98 mutations were previously unreported in the peer‐reviewed literature and variant databases.

The inheritance of those mutations in our positive cases (See Table 4) were autosomal dominant (AD) (*N* = 21 [29.17%, 21/72]), autosomal recessive (AR) (*N* = 34 [47.22%, 34/72]), and X‐linked (*N* = 15 [20.83%, 15/72]), respectively. The majority of the variants in AD diseases was de novo (57.14%, 12/21), defined as mutations present in the proband and not in the parents; while inherited ones were observed in four patients (19.05%, 4/21). Among the diagnosed patients with AR diseases, 27 patients had compound heterozygous variants and seven had homozygous variants. The two patients with X‐linked disorders had de novo mutations; 11 were inherited from his carrier mother (Table 4).

**Table 4:** Summary of the positive molecular diagnoses provided by NGS methods

Category	Number (%) of diagnoses
Autosomal dominant^a^	
De novo	12 (16.66%) [2]
Inherited	4 (5.56%)
Inherited unknown	5 (6.94%) [2]
Autosomal recessive^a^	
Compound heterozygous	27 (37.50%)
Homozygous	7 (9.72%) [1]
X‐linked hemizygous^a^	
De novo	2 (2.78%)
Carrier mother	11 (15.28%) [6]
Carrier mother (mosaic)	2 (2.78%)
Isolated cases	1 (1.39%)
Mitochondrial inheritance	1 (1.39%)
Total	72

Note

Number in brackets indicates cases with large copy number variant findings.

a Causal variants are point variants, small indels, inserts, or large exon indels, duplicates.

### 3.3 Effect of clinical presentation on molecular diagnosis

Approximately 24 of the 72 diagnosed individuals (33.33%, 24/72) have atypical or unrecognized infantile presentation of genetic disorders. Some examples include that of a 3‐month‐old infant with seizures that were caused by a pathogenic *ABCD1* (MIM 300371) variant, and a short‐limbed neonate hospitalized of persistent hyper‐lactic acidemia due to a defect in *COL2A1* (MIM 120140). Some other examples of atypical presentation in infants of known Mendelian disorders include minicore myopathy with external ophthalmoplegia, which is instantiated by an 8‐month‐old girl harboring *RYR1* (MIM 180901) mutations, who shows poor intermittent feeding, diffuse muscle weakness, and a *CHD7* (MIM 608892) mutation presenting only a facial asymmetry without heart defect, extremity abnormalities, and genital hypoplasia, such as identified in a 20‐day neonate.

To assess whether specific clinical presentations were more likely to be associated with a molecular diagnosis, the diagnostic rate was compared among patients who were annotated with different phenotypes as represented by HPO term. Analyses were performed at the top‐level branching of HPO phenotypes to ensure adequate counts of participants (Table 5). Individuals with phenotypes of HPO category “abnormality of metabolism/homeostasis” (HP: 0001939) were found to yield higher diagnostic rate, though insignificantly (odds ratio, 1.83; 95% CI, 0.56–6.04; *p* = 0.32). Otherwise, individuals without a definite HPO term were found to be significantly underrepresented in cases with atypical symptoms (odds ratio, 0.08; 95% CI, 0.01–0.73; *p* = 0.03).

**Table 5:** Comparison of diagnostic rate by NGS tests in groups with and without the phenotype

HPO term	HPO ID	Diagnostic rate in individuals with the term	Diagnostic rate in individuals without the term	Odds ratio (95% CI)	*p*
Abnormality of the blood and blood‐forming tissues	HP:0001871	2/3	70/95	0.71 (0.06–8.22)	0.79
Abnormality of the cardiovascular system	HP:0001626	0/2	72/96	0.33 (0.04–2.50)	0.28
Abnormality of the digestive system	HP:0025031	4/5	68/93	1.47 (0.16–13.80)	0.74
Abnormality of the ear	HP:0000598	2/3	70/95	0.71 (0.06–8.22)	0.79
Abnormality of the eye	HP:0000478	6/8	66/90	1.09 (0.21–5.78)	0.92
Abnormality of the genitourinary system	HP:0000119	2/3	70/95	0.71 (0.06–8.22)	0.79
Abnormality of the immune system	HP:0002715	6/8	66/90	1.09 (0.21–5.78)	0.92
Abnormality of the integument	HP:0001574	4/7	68/91	0.45 (0.09–2.17)	0.32
Abnormality of the metabolism/homeostasis	HP:0001939	18/22	54/76	1.83 (0.56–6.04)	0.32
Abnormality of the nervous system	HP:0000707	14/23	58/75	0.62 (0.23–1.62)	0.33
Abnormality of the skeletal system	HP:0000924	3/4	69/94	1.09 (0.11–10.94)	0.94
Abnormality of the endocrine system	HP:0000818	0/1	72/97	0.69 (0.06–7.99)	0.77
Abnormality of prenatal development or birth	HP:0001197	0/3	72/95	0.21 (0.03–1.35)	0.10
Abnormality of the musculature	HP:0003011	1/1	71/97	0.18 (0.02–2.11)	0.17
*N* a	**‐**	1/5	71/93	0.08 (0.01–0.73)	0.03*

Note

Abbreviations: HPO, human phenotype ontology; HP, human phenotype.

a Patients had clinical features of more than two of the broad aforementioned HPO term or atypical symptoms so that they were not given the exact HPO terms for their primary phenotypes.

### 3.4 Negative cases

Of 26 infants who did not receive a diagnosis in this study (Table 3): only one variant was observed in four infants (15.38%, 4/26) with a suspected compound heterozygous model; one infant received a partial diagnosis by a special panel, the variant (c.817C>T (p.Q273X) in *ATP13A4* (MIM 609556) gene that is predicted as a null allele explains several of the clinical features (seizures and epilepsy) of the patient; two infants (7.69%, 2/26) received a dual or triple molecular diagnoses respectively; among five infants (19.23%, 5/26), their previously unreported findings were predicted as either consistently nondamaging or inconsistent between two tools; for the other 14 individuals (53.85%, 14/26), no pathogenic variants related to patient phenotypes were identified in the analyzed genes.

## 4 DISCUSSION

While applying NGS to the diagnoses of 98 unrelated patients in their first year of life at a single tertiary institution, we observed an overall molecular diagnostic yield of 73.47%, which is higher than the positive rates of published clinical NGS reports (Okazaki et al., 2016; Smith, Willig, & Kingsmore, 2015; Stark et al., 2016). This difference is likely due to the number of participants, the nature of their clinical problems, and the selection bias of diagnostic tools between our study and others (Al‐Shamsi et al., 2016; Okazaki et al., 2016). Moreover, significantly higher detection rates with TRS analysis have been shown in this study (OR: 0.24; 95% CI (0.08–0.70); *p*: 0.01), as well as in previous studies (Coene et al., 2018; Ponzi et al., 2018). All the 31 diagnosed infants with the 11 most common disorders in our cohort were observed through TRS analysis. Our high diagnostic yield demonstrates that the importance of distinct NGS strategies may be made available to address genetic diagnosis of a myriad of monogenic disorders and the effect of disease spectrum itself on the outcomes.

In our study, there were 22 patients with primary indication of infantile‐onset inborn errors of metabolism (IEM) (Rice & Steiner, 2016). For 18 of them, the reported pathogenic variants derived from the specific IEM panel were fully consistent with their clinical/biochemical (if available) features. For one patient with features of metabolic acidosis, recurrent hypoglycemia, poor‐feeding, and vomiting, the initial panel test did not identify any mutations, while a positive diagnosis by WES was received as a Combined oxidative phosphorylation deficiency‐23(COXPD23, OMIM 616198) (Kopajtich et al., 2014), one of the common causes of inborn errors in energy metabolism. Among these 22 individuals, 20 chose IEM panel and 2 WES. The results of this group indicated that abnormality of the metabolism/homeostasis underlined a substantial proportion of pediatric disease burden; a number of IEM have nonspecific biomarkers so that their diagnosis can be challenging depending on the traditional approaches, and a TRS analysis covering appropriate panel of genes has significant clinical utility for this group. Our results also illustrated that some variants not captured by one pipeline were indeed detected by the other (Jacob et al., 2018; Mori et al., 2017).

In our study, we applied WES rather than TRS to 17 patients mainly because the patients had nonspecific features and/or because a feasible TRS analysis was unavailable. The diagnosis was confirmed in eight of the patients. The definite diagnoses were Minicore myopathy with external ophthalmoplegia (OMIM 255320), the Strudwick type of spondyloepimetaphyseal dysplasia (OMIM 184250), CHARGE syndrome (OMIM 214800), Acrokeratosis verruciformis (OMIM 101900), Obesity with impaired prohormone processing (OMIM 600955), Combined oxidative phosphorylation deficiency‐23 (OMIM 616198), Niemann‐Pick disease type C1 (OMIM 257220), and Pseudovaginal perineoscrotal hypospadias (OMIM 264600). The success in these cases showed that there was not prior knowledge of the genetic condition in the patients since all cases were sporadic, of highly variable phenotypes and of variable severity. Eleven patients developed their clinical manifestations during neonatal period or early infancy (before 3 months of age), and 10 of them were critically ill babies in our NICU who required rapid comprehensive genetic reporting for both prognostication and clinical decision making. Our results supported the conclusion (Meng et al., 2017) derived from the study by Linyan Meng et al that the atypical and unrecognized presentation of genetic disorders that were observed in some young infants further challenged the traditional paradigm of tiered genetic testing in critical care units because the earlier the onset, the faster the progression and consequently the shorter the life span (Fitzgerald et al., 2015; Retterer et al., 2016). Since this work did not provide a cost‐effective analysis of various NGS tests, as compared with conventional tools, in our patients, it is unknown whether NGS would increase or decrease the cost potentially. Also, since this work did not provide management details and follow‐up investigations of those patients, it is yet unknown how much NGS testing could affect a personalized treatment for each patient. We hope to find these answers in research yet to set up.

Negative results for 26 cases in our study could be explained by various reasons. We applied WES to nine patients and various panels to the other 17 depending on our understanding of the function of various genes, and the primary indication of each patient. Fourteen individuals (53.85%, 14/26) were not identified with any pathogenic variants related to their clinical phenotypes. The main reasons might be that the causative gene was not included in the panel design and that the genes encoding proteins involved in the alteration of a specific biochemical marker/clinical phenotype are currently unknown or unrelated to human diseases. Nine patients had primary indication of abnormality of the nervous system, their highly heterogeneous phenotypes and puzzling paraclinical investigations might confuse the clinical orientation, leading to their negative results. For five infants in this group, their variants were previously unreported and predicted as either consistently nondamaging or inconsistent between two in‐silico tools, indicating them as negative cases, which signal probable determination bias. It is therefore essential for clinicians to understand the strengths and limitations of every molecular test in order to choose the appropriate one for each patient (Meng et al., 2017). Also, functional studies should be performed to assess the impact of those VUS on the corresponding genes (Bao et al., 2014).

Unusual combination of signs, symptoms, and biochemical phenotypes sometimes can confuse even expert clinicians and geneticists. Therefore, a HPO term was used to classify the primary disorder of our cohort. Clinical assessments of the effect of HPO phenotype analysis on our diagnostic yields indicated a significantly low success rate for patients with atypical clinical features (no exact HPO terms); this is the same as the conclusion derived from another study: compound phenotype was noted to yield a lower diagnosis rate compared with an isolated phenotype. On the other hand, HPO analysis determined a higher diagnostic rate, though insignificantly, for the “abnormality of the metabolism/homeostasis” phenotype, which mainly might be due to the sample size of our study. But in another study, a higher diagnostic rate was associated with the “abnormality of the musculature” phenotype (Meng et al., 2017). Even though diagnostic yield was low for patients with nonspecific or overlapping clinical phenotypes, the confirmed case of Prader‐Willi syndrome is a good example of the application of NGS technology, because using traditional methods proved to have limited results with huge cost and lengthy duration for this disease (Butler, 2017).

## 5 CONCLUSION

In our study, NGS tools identified pathogenic mutations in 73.47% of our cases, demonstrating that they are informative in a tertiary clinical setting for Mendelian disorders. Moreover, it is proven by our study that NGS is effective in identifying new variants in known diseases as well as widening the spectrum of phenotypes resulting from deleterious variations in known genes. Therefore, it will not be long to see NGS tool as a routine diagnostic test for many genetic conditions.

## CONFLICT OF INTEREST

The authors declare neither conflict of interest nor financial interests.
